# Alternative insecticides for larval control of the dengue vector *Aedes aegypti* in Lao PDR: insecticide resistance and semi-field trial study

**DOI:** 10.1186/s13071-018-3187-8

**Published:** 2018-12-03

**Authors:** Sébastien Marcombe, Somsanith Chonephetsarath, Phoutmany Thammavong, Paul T. Brey

**Affiliations:** Institut Pasteur du Laos, Ministry of Health, Vientiane, Lao PDR

**Keywords:** *Aedes aegypti*, Vector control, Temephos, *Bti*, Diflubenzuron, Pyriproxyfen, Spinosad, IGR, Laos, Dengue

## Abstract

**Background:**

The mosquito *Aedes aegypti* is the primary vector of several arboviruses, such as dengue, chikungunya and Zika, and represents a major public health problem in Southeast Asia. In Laos, where dengue is reemerging, several *Ae. aegypti* populations from the capital Vientiane have shown resistance to the organophosphate temephos, a commonly-used larvicide for public health interventions.

**Methods:**

Here, we tested the insecticide susceptibility of a wild larval population of *Ae. aegypti* against *Bacillus thuringiensis israelensis* (*Bti*), diflubenzuron, pyriproxyfen and spinosad. Residual efficacies of *Bti* (VectobacWG®), diflubenzuron (Killmos®) and temephos (Abate®) were then evaluated under simulated field conditions against the wild *Ae. aegypti* population.

**Results:**

The larval bioassays showed that the wild *Ae. aegypti* strain was moderately resistant to temephos and spinosad (resistance ratio, RR < 5) and fully susceptible to the other insecticides (RR = 1). The simulated field trial bioassays showed that all of the insecticides tested remained above the WHO acceptable larvicide threshold after 28 weeks.

**Conclusions:**

These results suggest that *Bti* and diflubenzuron may be promising alternative larvicides for controlling dengue vectors in water-storage containers in Laos, especially against *Ae. aegypti* populations, in which resistance to temephos has been detected.

## Background

Dengue is a vector-borne disease of public health importance in tropical and temperate regions of the world, with approximately 40% of the global population at risk. In Southeast Asia, dengue is threatening more than 1.3 billion people, representing 52% of the at-risk global population, and outbreaks have been occurring regularly across the region for several decades. In Laos, dengue is reemerging; the most important recent outbreak was in 2013 when 44,098 estimated cases were reported, including 98 deaths. As of October 2017, more than 9200 cases have been reported in Laos and the 2017 epidemic is still ongoing [1]. Since there is still no specific medication or effective vaccine available for dengue, control of the disease is mostly focused on targeting the vectors, *Aedes aegypti* and *Aedes albopictus* [2], by reducing mosquito densities through: (i) the elimination or protection of the potential breeding sites [3]; (ii) biological control using of larvae predators, such as small fishes or copepods [4–6], or entomopathogenic bacteria, such as *Bacillus thuringiensis var. israelensis* (*Bti*) [7]; or (iii) chemical control methods using insecticides for larval and adult mosquito control [3].

Despite the organized efforts to promote community participation in the dengue control programme since the 1990’s [8, 9], Laos continues to face challenges addressing this important public health issue transmitted by *Aedes* mosquitoes. During dengue epidemics, dengue control programmes rely on the use of pyrethroid insecticides (e.g. permethrin and deltamethrin) to reduce adult mosquitoes, but this method is not consistent nor fully efficient due to the limited number of qualified vector control personnel and the low budget allocated towards mosquito control. Therefore, Laos has relied on the use of the insecticide temephos (Abate® formulation) to reduce *Ae. aegypti* or *Ae. albopictus* larvae. This insecticide formulation is used to treat large water containers and is distributed throughout the country in places where dengue cases are reported, or as an active measure to treat known breeding sites that cannot be removed or protected.

It is possible that temephos resistance has spread in the country, as the insecticide has been used routinely since 1987 [8] and several bordering countries of Laos (Cambodia, Thailand and Vietnam) have reported temephos-resistant *Ae. aegypti* mosquitoes [10–13]. Laos vector control teams regularly report the inefficacy of the Abate® sachets in treated habitats; recent studies in Laos have shown resistance or reduced susceptibility to temephos in *Ae. aegypti* and *Ae. albopictus* populations from several provinces and Vientiane capital (Marcombe, personal communication), the most dengue-afflicted foci in the country [14]. Moreover, temephos continues to be under international scrutiny and deregulation for its toxicity against aquatic and non-target organisms [15]. Public health officials and scientists from around the world agree that new strategies in general, and new larvicides more specifically, are urgently needed to implement effective vector control operations in the field to combat dengue. In developing countries such as Laos, these new insecticides have to be cost-effective and environmentally friendly, particularly for the non-target organisms. The development of specific insecticide classes for mosquito control has been limited in previous decades, but new alternatives, primarily originating from the agricultural market, show promise in controlling *Aedes* mosquitoes [16].

The bacterial insecticide *Bti* represents a safe and efficient larvicide for mosquito control because of its fast killing effect and good toxicological profile [17]. After ingestion by larvae, *Bti* produce, by sporulation, solid parasporal crystal composed of insecticidal toxins (Cry and Cyt families) that have different modes of action but both result in disrupting the osmotic balance of the midgut cells of the mosquito larvae. Schnepf et al. [18] and Lacey et al. [17] described in detail the complexity of the mode of action of these Cyt and Cry toxin families. Diflubenzuron is an insect growth regulator (IGR) that disrupts chitin synthesis and deposition in mosquitoes, and has showed promising efficacy against several mosquito species, including *Ae. aegypti* [19–23]. Pyriproxyfen is a juvenile hormone mimic IGR that suppresses embryogenesis, metamorphosis and adult emergence in insects [20]. Pyriproxyfen showed interesting vector control properties against *Aedes* spp. in auto-dissemination studies [21, 22]. Spinosad is a bioinsecticide belonging to the naturalyte class, which are based on metabolites derived from the actynomycetale *Saccharopolyspora spinosa* (spinosin A and D) [23]. Spinosad acts on both the nicotinic acetylcholine and the γ-aminobutyric acid (GABA) receptors, causing paralysis and death because of increased excitation of the insect’s nervous system [24]. This insecticide has a favorable profile with low environmental persistence and low toxicity to non-target insects and fish [25, 26]. The larvicides cited above have different modes of action compared to temephos, and therefore represent interesting compounds for the management of insecticide resistance (IR) in *Ae. aegypti* populations in Laos. They are all recommended by the World Health Organization (WHO) for use as vector control in drinking water sources and containers, hence they are safe to humans [27–30].

In order to provide information on alternative insecticides to the vector control teams in Laos, we studied the resistance levels of one *Ae. aegypti* population from Vientiane (IPL strain) against *Bti*, diflubenzuron, pyriproxyfen and spinosad. We also evaluated the residual efficacy of *Bti*, diflubenzuron and temephos against the wild IPL strain in simulated field trials. Pyriproxyfen and spinosad were not tested because of delivery delays.

## Methods

### Mosquito strains

Two strains of *Ae. aegypti* were used for the laboratory and semi-field trials. The USDA strain originating from Florida, USA, maintained in colony for decades, and obtained from Kasetsart University (Faculty of Agriculture, Entomology Department), was used as the insecticide susceptible reference strain for the larval bioassays. This strain is free of any insecticide resistance mechanisms (e.g. knock-down and enzymatic resistance mechanisms). The second strain, an *Ae. aegypti* colony (IPL strain) was established from wild, field-caught mosquito larvae collected from ovitraps placed at the Institut Pasteur du Laos (IPL) in the Sisattanak district of Vientiane capital in Kao-gnot village (17.962684°N, 102.615035°E). Female adults obtained from the F0 generation were blood-fed on quail (*Coturnix japonica*) and their F1 or F2 progenies were used for the larval bioassays and simulated-field condition bioassays.

### Laboratory evaluation of insecticide resistance

Larval bioassays were carried out using technical grades of temephos, *Bti*, diflubenzuron, spinosad and pyriproxyfen, according to WHO guidelines [31]. These insecticides were purchased from Sigma-Aldrich (Singapore, Singapore), except for *Bti w*hich was purchased from Valent BioScience (Libertyville, IL, USA). The class name, the mode of action, and the active ingredient purity of each insecticide are given in Table 1. Bioassays were performed using late third- and early fourth-instar larvae of the field strains. For each bioassay, larvae of each strain were transferred to cups containing 99 ml of distilled water and 1 ml of the insecticide tested at the desired concentration. Five cups per concentration (25 larvae per cup) and 5 to 8 concentrations in the activity range of each insecticide were diluted in ethanol, except for *Bti*, which was diluted in distilled water. Control treatments consisted of the addition of 1 ml of ethanol to 99 ml of water (distilled water for *Bti* assays). For each insecticide, three replicates were implemented. Larval mortality was recorded after an exposure of 24 h to temephos, spinosad and *Bti*. Because of the delayed action of diflubenzuron and pyriproxyfen, larval and adult mortality was assessed every day until emergence. In these cases, larvae were fed with dry cat food at a concentration of 100 mg/l and dead larvae were removed daily, as dead larvae may contribute to mortality. For each bioassay, temperature was maintained at 27 °C, with a 12-h light: 12-h dark photoperiod.

**Table 1:** Description of the insecticides used for the larval bioassays and the semi-field test

Insecticide	Class group	Mode of action	Larval bioassay, active ingredient (%)	Formulation use for the simulated test (Dose)
Temephos	Organophosphate	Acetylcholinesterase inhibitor	95.6	Granules (1 mg/l)
*Bacillus thuringiensis israelensis* strain H14	Bacterial larvicide	Cell membrane destruction	37.4 (3000 ITU/mg)	Water dispersible granules (8 mg/l)
Diflubenzuron	Insect growth regulator	Chitin biosynthesis inhibitor	98.1	Tablets (0.25 mg/l)
Pyriproxyfen	Insect growth regulator	Juvenile hormone mimics	99.10	Not tested
Spinosad	Naturalyte	Nicotinic acetylcholine receptors	97.6	Not tested

### Simulated field trial

The trial was carried out in Vientiane, Laos between October 2014 and May 2015, on the premises of the IPL. The effects of temephos (1 mg/l, Abate®, Bangkok, Thailand), diflubenzuron (0.25 mg/l, Killmos®, Bangkok, Thailand) and *Bti* (8mg/l, Vectobac®, Libertyville, USA) formulations were evaluated and compared against *Ae. aegypti* larvae from IPL strain at the dosage recommended by the WHO or the manufacturers for the control of mosquito larvae (Table 1). Blue plastic containers with a capacity of 200 l were used because they are a common container for water storage in Vientiane City and have been shown to be an important productive breeding habitat for *Ae. aegypti* (Marcombe, personal communication). These drums were filled up with 175 l of domestic water then covered with a mosquito net to prevent oviposition by wild female mosquitoes in the area and escape by adult mosquitoes introduced as larvae prior to counting. The containers were placed under a shelter to prevent direct exposure to rain and sunlight. Twelve containers (three replicates per insecticide) were allocated to insecticides at random. Three were left untreated and used as a control. Groups of 100 third-instar larvae of the F1 generation of the IPL strain were added to each container with 1 g of food (dry cat food) at time 0, and then every 10 days. The containers were replenished every 10 days to maintain the initial level of water. Emerging adults were collected from each container by using electric aspirators and then stored at -80 °C. Temperature and pH were checked every 10 days with a portable tester to detect any differences between replicates and/or treatments. External temperature and humidity were recorded by using a meteorological unit.

### Statistical analysis

For the determination of the intrinsic activity of each larvicide, three replicates with larvae from different rearing batches were made at different periods and the results were pooled for analysis (*n* = 300 larvae per dose). According to the WHO guidelines [31], test with control mortality > 5% have to be corrected using Abbott’s formula [32] and tests with control mortality > 20% have to be discarded. Results were analyzed using the log-probit method of Finney [33] using the Log dose Probit software (LdP) Line (Ehabsoft, Cairo, Egypt) to estimate the slope of regression lines and determine the 50% and 95% lethal concentration (LC_50_ and LC_95_, respectively) with 95% confidence intervals (CIs). The USDA and IPL strains were considered to have different susceptibilities to a given insecticides when their resistance ratio (RR) between their LC_50_ or LC_95_ (resistance ratio, RR_50_ or RR_95_) had CIs excluding the value of 1. Following the WHO criteria, the IPL strain was considered resistant if the RR_50_ is over 10, moderately resistant with a RR_50_ between 2 and 10 and susceptible if the RR_50_ is under 2 [31].

Regarding the simulated field trial, emergence inhibition rates (% EI) and 95% CIs were calculated for the average of the three replicates per insecticide according to the formula:

$$ \% EI=\left(\frac{C-T}{C}\right)\times 100 $$

where C is the emergence in the control and T is the emergence in the treated container at the same time period. For each formulation, curves were presented until the % EI decreased to < 80%, which corresponded to the threshold generally considered for reapplication of the treatment [31].

## Results

### Insecticide resistance larval bioassays

Results of the larval bioassays performed on the *Ae. aegypti* susceptible strain (USDA) and the wild strain (IPL) are shown in the Table 2. There was no control mortality over 5% so no correction with Abbot’s formula was needed. The IPL strain was fully susceptible to the insecticides *Bti*, diflubenzuron and pyriproxyfen with RR_50_ of 0.84, 1.1 and 0.2, respectively. The IPL strain was also susceptible to both temephos and spinosad but showed higher RR_50_’s between 2–5. The two IGRs (pyriproxyfen and diflubenzuron) had the highest insecticidal efficacy against the wild IPL strain, with an LC_50_ below 2 μg/l, compared to temephos, *Bti* and spinosad with an LC_50_ of 6.6, 11.8 and 69 μg/l, respectively.

**Table 2:** Resistance status of *Aedes aegypti* (USDA and IPL strains) against temephos and potential alternative insecticides. Numbers in bold correspond to a resistance ratio (RR) statistically different from 1. RR between 2 and 10 show a moderate resistance to the insecticide according to WHO criteria

Larvicide	Strain	No. of larvae	LC_50_ (95% CI) (μg/l)	LC_95_ (95% CI) (μg/l)	RR_50_	RR_95_	*χ* ^2^	*P*	Slope ± SE
*Bti*	USDA	1501	14 (12–21)	54 (31–199)	–	–	0.2	0.89	2.8 ± 0.6
	IPL	1600	11.8 (11.3–12.3)	21 (19–23)	0.8	**0.4**	5.5	0.24	6.6 ± 0.5
Diflubenzuron	USDA	1500	1.7 (1.5–1.8)	5.6 (4.6–7.2)	–	–	8.5	0.075	3.1 ± 0.3
	IPL	804	1.8 (1.4–2.1)	4.1 (3.8–6.8)	1.1	0.7	20.0	0.0005	4.6 ± 0.3
Pyriproxyfen	USDA	1500	0.086 (0.05–0.1)	0.049 (0.03–0.06)	–	–	4.4	0.11	1.4 ± 0.3
	IPL	699	0.019 (0.017–0.022)	0.098 (0.074–0.12)	**0.2**	**2.0**	7.6	0.055	2.4 ± 0.2
Spinosad	USDA	1500	14 (12–19)	40 (26–96)	–	–	0.4	0.79	3.6 ± 0.3
	IPL	1472	69 (62–77)	206 (170–270)	**4.9**	**5.2**	3.4	0.18	3.4 ± 0.3
Temephos	USDA	1250	2.9 (2.7–3.1)	6.6 (5.8–7.6)	–	–	3.7	0.59	4.6 ± 0.3
	IPL	2600	6.6 (6.2–6.9)	11.6 (10.5–13.5)	**2.3**	**1.8**	5.3	0.07	6.6 ± 0.5

*Abbreviations*: *USDA* Susceptible reference strain, *IPL* Wild strain, *LC* Lethal concentration, *CI* Confidence interval, *RR* Resistance ratio = LC of IPL/LC of USDA strain; *SE* Standard error

### Semi-field trial experiment

**Fig. 1:**
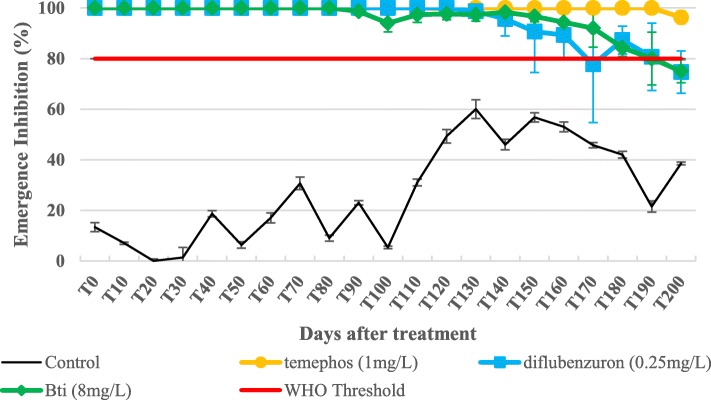
Emergence inhibition rates of *Ae. aegypti* mosquitoes (IPL strain) in the control and treated containers in semi-field trial

**Fig. 2:**
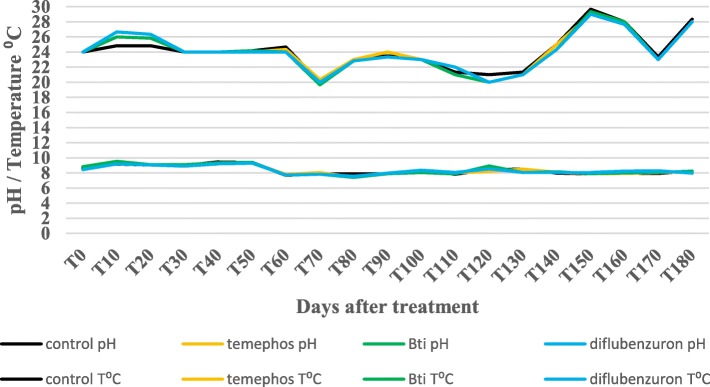
Average temperatures and pH in the control and treated containers

All treatments were effective (EI > 80%) for at least 22 weeks and temephos had the best efficacy of all treatments with 96% EI (4% emergence) after 28 weeks (Fig. 1). Twenty-eight weeks after the treatments, the average % EI in the containers treated with *Bti* was less than 80%, which is the limit recommended for a new treatment in field conditions. This threshold was exceeded in the containers treated with diflubenzuron after 24 weeks, but went back over 80% EI the week after, and dropping below 80% EI again at 28 weeks. The dramatic decrease of emergence in the control containers, dropping from 80% to 40% between T110 and T150, corresponds to an increasing temperature inside the containers during the same period (Fig. 2). During this period, the temperatures inside the containers rose from 22 °C to almost 30 °C. This event may have had consequences on the treated containers, delaying the increase of emergence for a few weeks. During the experiment, the average pH measured in the containers remained between 7.6– 9.2 (Fig. 2).

## Discussion

The objectives of this study were to evaluate candidate insecticides and to validate them under simulated field conditions. The specific modes of action of these insecticides represent valuable tools for future use in public health programmes in Laos. The larval bioassays showed that the wild IPL strain was fully susceptible against *Bti*, diflubenzuron and pyriproxyfen, and tolerant to spinosad. The ranges of insecticidal activities measured in our study were comparable with other studies tested against *Ae. aegypti* in several countries [34–39]. The absence of resistance against these insecticides by the wild IPL strain is encouraging, and anticipated, since these insecticides have never been used in Laos for vector control operations.

The insecticide temephos had the best residual efficacy compared to the other insecticides. This can be explained by the dose used to treat the containers, which is much higher than the LC_95_ of the wild IPL strain, but the efficacy would probably have not been as long against the Laos resistant strains found throughout the country. The synergistic effect of the *Bti* toxins together may explain why no resistance has been detected yet in areas where this bio-insecticide has been used routinely for years [17, 39], theoretically making it a good alternative for *Aedes* control [34, 35]. The IGRs diflubenzuron and pyriproxyfen represent other potential alternatives to decrease local mosquito population abundance. However, the use of IGRs should be implemented with caution, with recent studies showing evidence of resistance to IGRs developing in *Aedes* sp. field populations [34, 39–41]. Despite no cross-resistance between IGRs and conventional insecticides demonstrated in *Aedes* mosquitoes, Yunta et al. [42] showed the involvement of P450 monooxygenases (i.e. insecticide detoxifying enzymes) in the cross-resistance mechanisms between pyriproxyfen and pyrethroids in the malaria vector *Anopheles gambiae* in Africa. Importantly, overexpressed P450s genes are responsible for pyrethroid resistance in *Ae. aegypti* mosquitoes [43] and have been reported worldwide [12, 44]. A recent biochemical study showed that several pyrethroid resistant *Ae. aegypti* populations from Laos had higher levels of P450s enzymes compared to the susceptible reference strain USDA. The tolerance of the IPL strain to spinosad (RR > 5) could be explained by the differences in genetic background between the wild and susceptible strains, the later having been colonized for decades with no exposure to insecticides or other xenobiotics [45]. Furthermore, the spinosins (A and D) act on different targets of the mosquito nervous system, lowering the occurrence of cross-resistance with conventional insecticides [38].

The new candidate insecticides *Bti* and diflubenzuron showed equal efficacy as temephos for up to 7 months in simulated field conditions. The result of *Bti* measured is correlated to a study implemented in similar conditions in the Martinique (Caribbean) [34]. However, in that study, the temephos efficacy (EI > 80%) was 20 weeks, compared to 28 weeks in Laos. This can be explained by the high temephos resistance levels measured in this *Ae. aegypti* population compared to the IPL strain (i.e. RR_95_ of 175 and 1.76, respectively). Other simulated field studies measured the efficacy of different diflubenzuron formulations (granular sand and tablet formulations) and the results showed EI > 80% for more than 23 weeks against *Ae. aegypti* [46, 47], making this insecticide a good candidate for control. The rotational or mosaic use of *Bti*, diflubenzuron and temephos in Laos may be a good strategy to prevent or limit insecticide resistance development.

*Bti* is extensively used for the control of mosquito species [48], but compared to other insecticides, such as temephos and the alternatives diflubenzuron and spinosad, it has short-term residual efficacy in natural conditions against *Ae. aegypti*. In large-scale field trials implemented in Martinique and the Philippines, *Bti* formulations used to treat large water containers were effective only 3–4 weeks after treatments, while spinosad and diflubenzuron were effective for 16 weeks [34, 49]. Thavara et al. [47] also showed a good residual activity of diflubenzuron under field conditions, with 23 weeks efficacy. Marcombe et al. [34] showed that, similar to *Bti*, pyriproxyfen lost its efficacy after four weeks post-treatment. The short residual activity of an insecticide is a concern for their use in dengue control programmes because operators have to repeat the treatments regularly [50]. The use of a combination of two insecticides is a way to increase the efficacy of the treatments. For example, combination of *Bti* and *Lysinibacillus sphaericus* (*Lsph*) compensates for the low residual efficacy of the former by the synergistic effect of their toxins. Another study showed the synergistic effect of spinosad and pyriproxyfen in mixture against *Ae. aegypti* in a large-scale field trial [51]. The residual efficacy of the combination was increased by 1.5 months, in comparison of the insecticides alone. Recent laboratory studies showed that octopamine receptor agonists (OR agonists) increased the potency of IGRs against *Ae. aegypti* [52]. The use of OR agonists in combination with IGR is a promising tool for vector control but more studies have to be implemented to understand the synergistic mechanisms involved.

The good residual efficacy of the diflubenzuron and *Bti* formulations is encouraging, however, the results observed in well-controlled conditions has to be taken with caution and cannot be used to predict the performance of the formulations under field conditions. Indeed, in real treatment conditions, the direct exposure to sunlight, rain and organic matter and water exchange practices can significantly reduce the residual efficacy of an insecticide [34, 47, 53]. Despite the relatively good residual activity of the temephos formulation observed against the wild IPL strain, *Aedes* control teams in Laos continue to report temephos use failures in treated containers. These examples emphasize the need for alternatives to temephos, and the continued use of this insecticide in Laos should be reconsidered, as it will certainly lead to fully resistant *Aedes* populations.

## Conclusions

This study measured the IR status of a wild *Ae. aegypti* population from Vientiane, under laboratory conditions, against the conventional insecticide temephos and candidate larvicidal alternatives to better inform Laotian dengue control teams. Insecticide resistance bioassays showed that the wild IPL field strain was susceptible to *Bti*, diflubenzuron and pyriproxyfen, making them good alternative insecticides to temephos for vector control. Temephos, *Bti* and diflubenzuron showed good residual efficacy against the wild IPL strain in widely used plastic water container habitats in simulated, controlled field trials; however, a more comprehensive study to measure the real efficacy of these alternatives in the environmental and socio-economic conditions of Laos should be pursued. If these insecticides are to be used in the future in Laos, constant monitoring of the insecticide resistance status of *Aedes* populations is recommended. Furthermore, because of the increasing importance of these alternative insecticides for vector control, the study of the insecticide resistance mechanisms in mosquitoes against alternatives should not be neglected [44].

## Abbreviations

GABA: γ-aminobutyric acid
*Bti*: *Bacillus thuringiensis israelensis*
CI: Confidence interval
EI: Emergence inhibition
IGR: Insect growth regulator
IPL: Institut Pasteur du Laos
IR: Insecticide resistance
LC: Lethal concentration
*Lsph*: *Lysinibacillus sphaericus*
OR: Octopamine receptor
RR: Resistance ratio
SE: Standard error
